# Digital Interventions to Modify Skin Cancer Risk Behaviors in a National Sample of Young Adults: Randomized Controlled Trial

**DOI:** 10.2196/55831

**Published:** 2024-07-02

**Authors:** Carolyn J Heckman, Anna Mitarotondo, Yong Lin, Olga Khavjou, Mary Riley, Sharon L Manne, Amy L Yaroch, Zhaomeng Niu, Karen Glanz

**Affiliations:** 1 Rutgers Cancer Institute New Brunswick, NJ United States; 2 Rutgers School of Public Health Piscataway, NJ United States; 3 RTI International Research Triangle Park, NC United States; 4 Medtronic Denver, CO United States; 5 Gretchen Swanson Center for Nutrition Omaha, NE United States; 6 Rutgers School of Health Professions Piscataway, NJ United States; 7 University of Pennsylvania Philadelphia, PA United States

**Keywords:** skin cancer prevention, sun protection, sun exposure, digital intervention, social media, young adults, sun, skin cancer, prevention, cancer, randomized controlled trial, Facebook, Instagram, engagement, behavior, protection, skin, sunscreen

## Abstract

**Background:**

Young adults engage in behaviors that place them at risk for skin cancer. Dissemination of digital health promotion interventions via social media is a potentially promising strategy to modify skin cancer risk behaviors by increasing UV radiation (UVR) protection and skin cancer examinations.

**Objective:**

This study aimed to compare 3 digital interventions designed to modify UVR exposure, sun protection, and skin cancer detection behaviors among young adults at moderate to high risk of skin cancer.

**Methods:**

This study was a hybrid type II effectiveness-implementation randomized controlled trial of 2 active interventions, a digital skin cancer risk reduction intervention (UV4.me [basic]) compared with an enhanced version (UV4.me2 [enhanced]), and an electronic pamphlet (e-pamphlet). Intervention effects were assessed over the course of a year among 1369 US young adults recruited primarily via Facebook and Instagram. Enhancements to encourage intervention engagement and behavior change included more comprehensive goal-setting activities, ongoing proactive messaging related to previously established mediators (eg, self-efficacy) of UVR exposure and protection, embedded incentives for module completion, and ongoing news and video updates. Primary outcome effects assessed via linear regression were UVR exposure and sun protection and protection habits. Secondary outcome effects assessed via logistic regression were skin self-exams, physician skin exams, sunscreen use, indoor tanning, and sunburn.

**Results:**

The active interventions increased sun protection (basic: *P*=.02; enhanced: *P*<.001) and habitual sun protection (basic: *P*=.04; enhanced *P*=.01) compared with the e-pamphlet. The enhanced intervention increased sun protection more than the basic one. Each active intervention increased sunscreen use at the 3-month follow-up (basic: *P=.*03; enhanced: *P*=.01) and skin self-exam at 1 year (basic: *P=.*04; enhanced: *P*=.004), compared with the e-pamphlet. Other intervention effects and differences between the Basic and Enhanced Intervention effects were nonsignificant.

**Conclusions:**

The active interventions were effective in improving several skin cancer risk and skin cancer prevention behaviors. Compared with the basic intervention, the enhanced intervention added to the improvement in sun protection but not other behaviors. Future analyses will explore intervention engagement (eg, proportion of content reviewed).

**Trial Registration:**

ClinicalTrials.gov NCT03313492; http://clinicaltrials.gov/ct2/show/NCT03313492

## Introduction

### Skin Cancer Risk

Almost 5 million Americans are treated for skin cancer annually, and its incidence is rising [1-5]. Additionally, keratinocyte carcinomas can be a chronic disease, requiring ongoing costly treatments and resulting in compromised quality of life similar to some other cancers [6]. Although largely preventable, skin cancers, particularly melanomas, can be deadly, debilitating, damaging to tissues and organs, and disfiguring. Risk factors for skin cancers include personal or family history of melanoma or keratinocyte carcinomas, certain phenotypic (eg, fair skin) and other physical characteristics (eg, numerous moles) [7-19], as well as excessive UV radiation (UVR) exposure from the sun or tanning devices [20-25]. Most skin cancers are preventable with sun protection such as minimizing UVR exposure and wearing protective clothing and sunscreen [26,27]. Early detection via skin examination by an individual, partner, or health care provider may also help improve treatment outcomes and reduce morbidity and mortality [28].

Young adulthood, defined here as ages 18 to 25 years, is an important window for skin cancer risk reduction interventions. Melanoma is one of the most common cancers in people younger than 30 years [29]. Adolescents in the United States have the lowest sun protection rates of all age groups [30] and engage in increased UVR exposure as they move into adulthood [31]. This time period represents a critical period in forming sun protection habits because only 25% of lifetime UVR is accumulated by age 18 years [32]. Skin cancer risk behaviors, including lack of sun protection, sunburns, and indoor tanning, peak around age 25 years [33,34]. Unfortunately, young adults tend to be resistant to preventive health recommendations because, as a group, they perceive themselves as having more immediate priorities than disease prevention and believe that the consequences of their current health behaviors are in the distant future [35-37]. In addition, young adults are more likely to engage in risky behaviors and can be more influenced by their peers than older adults [35-37]. Prior studies of behavioral interventions to increase sun protection or decrease UVR exposure among healthy young adults in the United States [38-45] have been limited in their reach or duration of assessment.

### Social Media and Digital Intervention

Social media has been almost universally adopted by young adults. In 2021, almost all 18-29 year olds used the internet [46], approximately 96% owned a smartphone [47], and approximately 70% used Facebook and Instagram [48]. Social media recruitment can be efficient [49-52], and digital behavioral interventions can be disseminated widely and be cost-effective to maintain [53,54]. Digital interventions designed to improve health behaviors (eg, exercise and weight loss) in various populations have been found to produce medium effect sizes and consistently outperform similar nondigital interventions [54], but longer-term outcomes have been minimally evaluated for skin cancer prevention digital interventions [55].

Given the potential of digital behavioral health approaches, we developed an individually tailored, interactive, multimedia, and theoretically grounded (Integrative Model of Behavioral Prediction [IM]) web-based intervention targeted to young adults (basic) [56,57]. We evaluated the intervention in a large sample of 18-25 year olds (n=964) at moderate to high risk of developing skin cancer, recruited from a consumer research panel hosted by a market research company, in a randomized controlled efficacy trial. Behavioral risk factors for melanoma were significantly improved. Intervention effect sizes (Cohen *d*) were 0.53 for sun protection and 0.43 for UVR exposure behaviors at the 3-month follow-up compared with an assessment-only condition [58]. Sunburns were also significantly reduced. This intervention is the basic intervention for the study described in this paper.

### Study Purpose

Although the results of the efficacy trial were promising, we proposed that the intervention and outcomes could be further improved. Our goal was to improve engagement with and impact of the Basic Intervention on sun protection, UVR exposure, and engagement in skin cancer examination. To improve effects of the basic intervention, we added several key interactive features and strategies suggested by prior participants, our data, and supported by the literature (ie, by creating a mobile version, adding incentives embedded in the intervention, an enhanced goal-oriented feature, ongoing email and SMS text messages related to previously identified mediators of behavior change, and ongoing news and video updates).

To assess the potential for future dissemination, we conducted a hybrid type II effectiveness-implementation trial [59], which has the goal of increasing efficiency of moving novel evidence-based interventions to the community through mass access and reach. The purpose of this study was to implement the enhanced intervention (enhanced) with young adults aged 18-25 years at moderate to high risk of developing skin cancer and evaluate the intervention’s effectiveness in a sample recruited via the web through national dissemination to a general population. We hypothesized that effectiveness and longer-term maintenance effects on UVR and sun protection outcomes would be best for enhanced, second for the original basic condition, and worst for an electronic pamphlet (e-pamphlet) control group. To our knowledge, these are the first entirely digital behavioral skin cancer prevention interventions targeted to healthy young adults recruited from the community that have been evaluated in a national longitudinal randomized controlled trial (RCT) in the United States. If found to be effective, these interventions could be feasibly scaled up for widespread delivery, with the potential to reduce skin cancer risk in a large population of US young adults.

## Methods

### Procedures

This study assessed 2 digital skin cancer risk reduction programs compared with a control condition for young adults in the United States between the ages of 18 and 25 years (ClinicalTrials.gov NCT03313492). Recruitment efforts are described in detail elsewhere [60]. Briefly, recruitment of study participants was conducted through paid advertising on Facebook, Instagram, and Twitter and unpaid social media posts between September 2018 and April 2019. Audience demographic and behavioral characteristics including age range, location (United States), and interests such as outdoor activities and physical fitness were identified to efficiently target the intended audience. Advertisement content focused on potential heath, appearance, and financial benefits of participation (eg, “Healthy skin is beautiful skin”). The objective of the advertisements was to encourage potential participants to click the call-to-action buttons such as “Sign Up” or “Learn More” that directed them to a study-specific landing web page or sign-up web page. The landing page included relevant images and brief information about the study and reasons why individuals might want to participate, including brief testimonials from prior participants. Individuals were then instructed to create an account on the sign-up web page with a phone number, email, and password. Once an individual indicated their interest and created a study account, they were automatically directed to complete a brief eligibility screener. If eligible, individuals were invited to complete the online informed consent form. After consent, participants were directed to the 10-minute online baseline survey. If enrollees completed the baseline survey, they were automatically directed to their intervention condition and received a US $5 electronic gift card. A computerized program was created to randomize participants on a 2:2:1 basis in blocks of 10 to either basic, enhanced (described below), or a noninteractive skin cancer prevention educational web page modeled after an e-pamphlet from the American Cancer Society [61]. Participants were invited by email, SMS text message, and telephone if necessary to complete online 10- to 15-minute follow-up surveys at 4, 12, 24, and 52 weeks.

### Ethical Considerations

This research adhered to appropriate ethical review and approvals as per institutional guidelines (Institutional Review Board #Pro2018001543). Participants provided informed consent electronically and were informed of their ability to opt out. Identifiable data were stored in password-protected files accessible only to approved research personnel. Data were deidentified for analysis. Participants were informed that the total study incentives would be up to US $120 for completing all 5 online surveys over the course of 12 months, plus periodic gift card raffles.

### Active Interventions

#### UV4.me (Basic)

The Integrated Model of Behavioral Prediction (IM) informed the development of the Basic Intervention, for example, by focusing on modification of attitudes, beliefs, perceived norms, and self-efficacy, as well as intentions and behaviors. The basic intervention, described elsewhere in more detail [56], is targeted to young adults, individually tailored, and includes interactive, multimedia, and goal-setting components. It includes 12 modules with content related to a specific topic important in terms of risk or protective behaviors: Why do people tan? To tan or not to tan? Indoor tanning, UVR & looks, UVR & health, Skin cancer, Skin damage, Sunscreen, Shade, Clothes, Skin exams, and Sunless tanning. Several more general sections (eg, My Stuff and Resources) are also included. Tailoring algorithms were created to direct participants to focus on certain modules based on their responses to a few initial questions (eg, the indoor tanning module was recommended for indoor tanners). Throughout the program, participants were asked questions and provided with tailored feedback (eg, “Do you know people who tan? If so, how likely are they to affect your choice to tan or not?”). A number of interactive elements (eg, videos and quizzes) were created to encourage participant engagement with the intervention. For example, at the end of each module, participants could choose to set a goal or not (eg, “I want to put sunscreen on most mornings”).

#### UV4.me2 (Enhanced)

New features and strategies were chosen based on participant feedback from the original UV4.me (eg, strategies suggested to make basic more interactive), our data (eg, number of mobile users who tried to access basic), and evidence-based literature and reviews and models of effective eHealth interventions [62,63] and implementation strategies [64-66] (Table 1). Several enhancements were expected to improve recruitment and representativeness, engagement, and behavioral outcomes:

1. Mobile site: In the original UV4.me study, the intervention was not mobile-friendly [56,58]. For the study described in this paper, both active interventions and the control condition were created using a responsive design, so that they were accessible via a variety of devices including mobile Android and Apple devices. It was thought that a mobile version could improve participant reach directly because more people would have access and may improve effectiveness indirectly by facilitating ongoing engagement.
2. Goal setting: Goal setting addresses several aspects of the IM, for example, intentions, environmental constraints, self-efficacy, and behavior. Users were given the option to commit to a behavioral goal relevant to each module (eg, “cut back on indoor tanning”). They were given tips on goal setting (eg, “set realistic goals”), asked to select from a list of potential reasons why the goal might be important to them (eg, “to avoid getting skin cancer”), strategies to assist them (eg, “check out sunless tanners”), note their progress (“great,” “not great,” “ok”) and challenges (open-ended comments from participants) over time, see their goals summarized on a page called MyGoals, and receive motivating email or SMS text messages (eg, “If you chose a goal, keep working on it”) and online feedback (eg, “Keep trying!”). Goal setting is a well-established empirically supported behavior change technique including for internet interventions [67,68]. Both basic and enhanced participants also received 4 automated goal-related messages with decreasing frequency over the year.
3. IM mediator messages: Email or SMS text messages were sent to participants based on IM constructs that were found to mediate UVR exposure and sun protection outcomes in the prior study, for example, knowledge and self-efficacy. Enhanced participants were sent 6 automated mediator messages with decreasing frequency over the course of the year, for example, “Plan ahead, and bring protection with you. Make it part of your routine. Encourage your friends to protect their skin too.”
4. Incentives: The enhanced site offered incentives in the form of clickable discount codes (eg, 5%-50% off) and links to sales websites of companies that sell sun protection products (eg, sunscreen, hats, and sunglasses) at the end of each of the 12 main content modules. Incentives can reinforce behavioral implementation, retention, and health behavior change, including in online trials, especially among those initially least motivated or for behaviors that are not intrinsically enjoyable (eg, applying sunscreen) [69-76].
5. Ongoing news updates and video library. Ongoing news and video updates were provided by adding new material (eg, videos, news and media stories, and new research summaries) to the enhanced intervention throughout the study (approximately once per month for 12 months). Updated information was a factor identified in a review or model of effective eHealth interventions [63], is important for dissemination of online interventions [64,65], and has been shown to increase intervention engagement [77,78].

**Table 1:** Basic and enhanced intervention features.

Intervention features			Basic		Enhanced
**Major content**					
	12 interactive, multimedia modules on topics such as skin cancer risk factors, sun protection, and indoor tanning	✓		✓	
	Some content tailored to participant demographics and skin cancer risk factors and behaviors				
	My Stuff repository of personalized goals and recommendations				
	Clickable URLs for skin cancer–related resource websites (eg, Skin Cancer Foundation and American Academy of Dermatology)				
**Goal setting**					
	*Basic*: prompts users to set behavior change goals (eg, related to sunbathing, indoor tanning, sunburns, and sunscreen use) *Enhanced*: prompts users to set behavior change goals, identify barriers, problem-solve, note progress, and receive motivating feedback (eg, “Great job, your tanning is going down over time!”), and reminders via email or SMS text messages	✓		✓	
**Video library**					
	Educational, news, and entertaining videos related to skin cancer prevention (updated at least monthly)			✓	
**News updates**					
	Timely news and media articles, new research, charity events, etc related to skin cancer prevention (updated at least monthly)			✓	
**Product discounts**					
	Unique coupon discount codes (5%-50% off) for sun protection products (eg, sunscreen, protective clothing, hats, and sunglasses) became available at the completion of each module			✓	
**Mediator messages**					
	Six email and SMS text messages were intermittently sent to participants related to theory-informed intervention effect mediators identified in the prior efficacy trial (eg, knowledge and self-efficacy)			✓	

### Control Condition: E-Pamphlet

A free online American Cancer Society pamphlet on skin cancer prevention and early detection [61] was used as the control condition and was distributed on a noninteractive web page.

### Measures

#### Eligibility Screener and Enrollment

**Figure 1:**
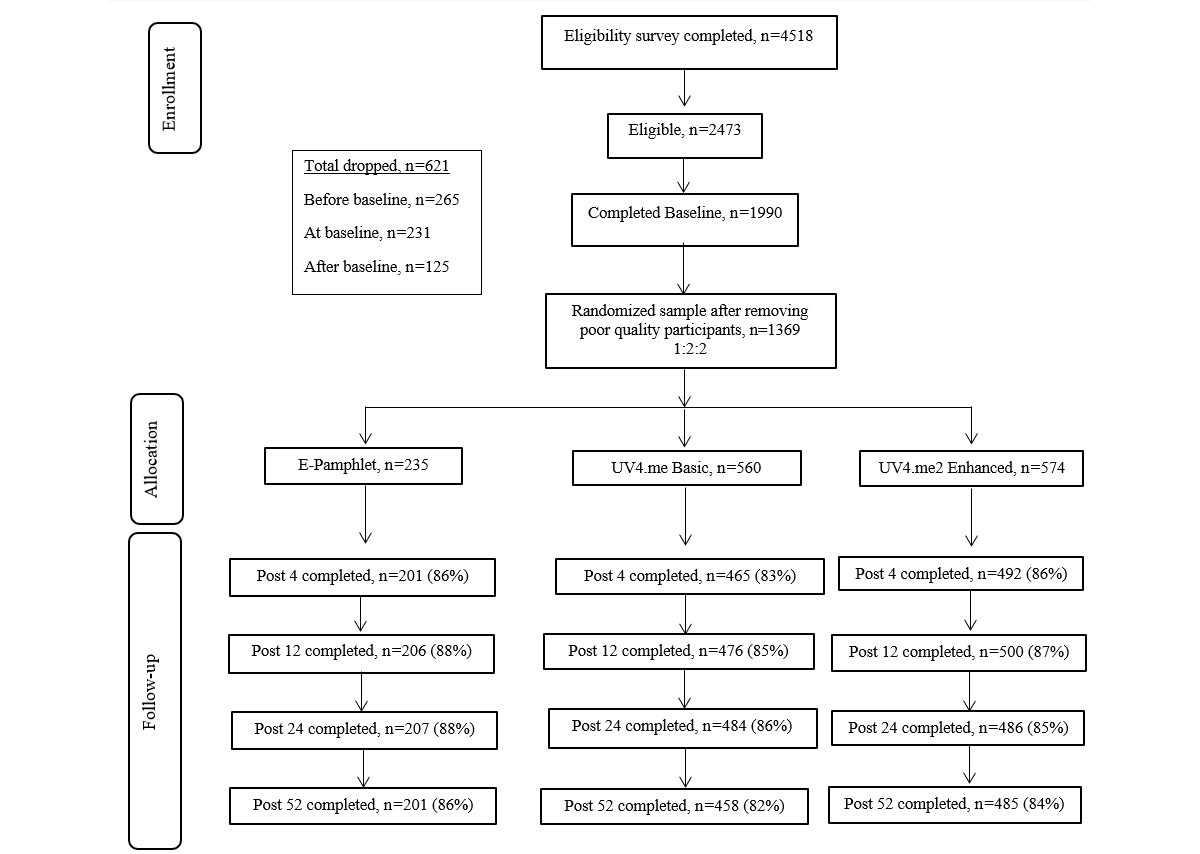
CONSORT (Consolidated Standards of Reporting Trials) flow diagram.

Participants eligible for the study were 18-25 years old, or 19-25 years old if in Alabama or Nebraska, for which 19 years is the age of legal majority. Additional criteria were English speakers, living in a US state or the District of Columbia, had at least weekly internet access, did not have a personal history of skin cancer, and did not report “always” protecting their skin from the sun when outdoors during warm weather. Participants also responded to 11 items related to phenotypic and behavioral risk factors (eg, fair skin and history of sunburns) that indicated moderate to high risk of developing skin cancer (ie, score of ≥27 on the Brief Risk Assessment Tool [BRAT]) [79]. The numbers of participants who were screened, eligible, and consented to participate in the study were assessed (Figure 1). Baseline completion date was noted.

#### Demographics

Demographic characteristics assessed were age, gender identity, sexual orientation, cohabitation status, race, ethnicity, student status, educational level, employment status, annual income, current climate, and zip code. Weekday time spent on the internet and personal time spent on a mobile device were also assessed using items adapted from the Health Information National Trends Survey [80].

#### Social Desirability

Social desirability was assessed using the 4-item Brief Social Desirability Scale (eg, “I would never lie to people”), which has demonstrated adequate internal reliability and validity among young adults [81]. Endorsing 3 or 4 of the items indicates high social desirability.

#### Attention Check

Attention to survey questions was assessed in baseline and all follow-up surveys using the attention check question: “How often in the LAST MONTH did you breathe when you were outside? Please choose ‘not applicable.’ This will verify your careful reading of this item” [82]. This was used as part of an overall data quality score (DQS) described below.

#### Skin Cancer–Related History

Additional items asked about history of skin cancer among first-degree relatives, the degree of tanning and burning that occurs after 1 week in the sun, and whether participants had received a full body skin cancer examination by a health care provider in the last 12 months [58].

#### Behavioral Outcomes

##### Primary Outcomes

Skin cancer–related behavioral outcomes were assessed at baseline and 4 follow-up time points: 4, 12, 24, and 52 weeks. We assessed *sun protection* frequency in the last month (eg, face and body sunscreen use, wide-brimmed hats, long-sleeve shirts, long-sleeve pants, sunglasses, and shade). Response options were 0=never to 4=almost always. Participants were asked how many days in the past month they experienced various types of *UVR exposure* (eg, sunburns, sunbathing, intentional and incidental sun exposure, indoor tanning, clothes that exposes skin to the sun, and tanning products). Summary indexes for sun protection and UVR exposure were created by averaging the items. Items were adapted from Glanz et al [83] and Ingledew et al [84] that our team has cognitively tested and assessed psychometrically with young adults [22].

We adapted the 4 items from the Habit Automaticity subscale of the Self-Report Habit Index to measure behavior patterns associated with the formation of a habit (eg, “Protecting my skin from UVR is something I do without thinking”) [85].

#### Secondary Outcomes

Specific items including sunburns and indoor tanning were considered secondary outcomes because sunburns and indoor tanning are unique risk factors for skin cancers, and these occur less frequently than sun exposure and protection in general. Participants were also asked about whether they had been screened for skin cancer by a health care provider or by themselves or a partner. The secondary outcome items were adapted from Glanz et al [83] and Ingledew et al [84] and cognitively tested and assessed psychometrically among young adults [22]. In terms of psychometric evidence for the outcome measures, several studies have demonstrated the reliability and validity of self-report questionnaires of UVR exposure, protection, and detection compared with observation and objective measures with no systematic bias identified among various populations including young adults [86-90].

#### Data Quality Score

As occurs in other internet-based and survey research, some participants may attempt to enroll more than once or provide inaccurate responses to be deemed eligible and earn study incentives [91-93]. Participants were excluded from this study for giving responses that were very likely of poor quality for issues such as providing a nonunique or nonworking e-mail address or phone number [91]. In addition to removing participants who appeared to attempt to enroll more than once or provided clearly poor-quality data, we used several additional items and responses to assess the quality of the remaining participants. These included responses to the attention check item, social desirability item, 2 items with potentially discrepant responses (ie, work hours and income), responses that were extremely similar to another participant submitted close in time, nonsensical open-ended responses, extremely short survey completion times, reporting on features only included in another condition, reporting that they had seen social media postings related to the study or program since enrolling, or reporting that they knew someone else in the study. Responses to each item were coded as “suspicious” or not. These codes were summed to create a DQS for each participant. Scores could range from 0 to 17, with higher scores indicating worse quality. Participants with a score of 9 or higher were excluded from data analyses. Scores from 0 to 8 were used to weight the outcome data so that higher quality data were given higher weights as described below. DQS scores and procedures were determined before outcome analyses.

### Analyses

#### Effectiveness

We compared demographics across randomization arms using χ^2^ tests for categorical variables and heterogeneous variance models for continuous variables to account for potential differences in variance across intervention arms at baseline. The primary outcomes were continuous and reassessed at weeks 4, 12, 26, and 52: sun protection, habitual sun protection, and UVR exposure, all in the last month. The secondary outcomes were binary and reassessed at the same time points as the primary outcomes: sunburn in the last month, indoor tanned in the last month, skin self-exam in the last 3 months, skin exam by a health care provider in the last 12 months, and sunscreen worn on body in the last month. Weighted multivariable mixed effect linear regression together with a robust error variance estimation method was used for analysis of the primary outcomes. The weight was a linear function of the DQS with a value of 1 if DQS=0 and a value of 1/2 if DQS=8. Post hoc analysis based on the model was used to compare the effectiveness of enhanced with basic and enhanced with the e-pamphlet. For analyses of the primary outcomes, the covariates included baseline value, recruitment season (eg, summer, fall, winter, and spring) since some of the behaviors are associated with changes in temperature, residential climate (northern vs southern and tropical), study arm, follow-up time point, and interaction of arm and time point. When the interaction was not significant, it was not included in the final model. For analyses of the secondary outcomes at each time point, covariate-adjusted risk difference estimation based on the logistic regression model proposed by Ge et al [94] was used. The covariates included baseline value, recruitment season (eg, summer, fall, winter, and spring), residential climate (northern vs southern and tropical), and study arm. All the tests were 2-sided with a significance level of 5%.

#### Missing Data

We also used multiple imputation (MI) methods for missing data to estimate the effectiveness of intervention as sensitivity analyses. MI was based on outcomes of interests and baseline variables: age, gender, race and ethnicity, sexual orientation, family history of skin cancer, level of education, income, climate, cohabitation, BRAT skin cancer total risk score, and number of hours in a day on the internet for personal reasons. A total of 15 imputed data sets were generated by the multivariate sequential regression approach, called the chained equations method [95], using IVEware software [96], and Rubin’s rule [97] was applied to combine results using SAS PROC MIANALYZE [98]. Because the results without MI and with MI were consistent, only results without MI are reported.

## Results

### Participant Characteristics

Online eligibility surveys were completed by 4518 individuals (Figure 1). Of these, 2473 (54.7%) were deemed eligible. The main reason for being ineligible was a BRAT skin cancer risk score denoting lower risk (1056/4518, 23.4%, of individuals screened not meeting criteria), followed by age other than 18-25 years (767/4518, 17%, not meeting criteria) or self-reporting “always” engaging in sun protection behaviors outside (279/4518, 6.2%, not meeting criteria). Of these, 80.5% (1990/2473) submitted online baseline surveys. The study team dropped 13.7% (621/4518) of potentially eligible participants for attempting to enroll more than once or for poor data quality. This figure is lower than our prior study (22%) [58] but higher than the goal of 5% being careless responders. DQS ranged from 0 to 17 (worse quality), with a median (IQR) score of 3.00 (1.00-5.00) and a mean (SD) score of 3.63 (3.01). Of the 1369 who were randomized to 1 of the 3 intervention conditions, the proportion of completed surveys at follow-up was 1158 (84.6%) at 4 weeks, 1182 (86.3%) at 12 weeks, 1177 (86.0%) at 26 weeks, and 1144 (83.6%) at 52 weeks. These completion rates were similar across the 3 intervention conditions.

At baseline, participants’ average age was 22.3 (SD 2.31) years (Table 2). Approximately 69% (949/1369) of the sample was female, 83% (1141/1369) White non-Hispanic, 73% (999/1369) heterosexual, 34.3% (470/1369) had at least a college education, 47% (649/1369) earned ≤US $15,000 in the last year, 75% (1021/1369) lived in a state with a northern climate (consistent with classifications of states by climate), and over 87% (1185/1369) lived with someone part or all of the last year. There were no significant differences in these demographic variables across the intervention conditions (*P* values ≥.13), suggesting successful randomization.

**Table 2:** Participant demographic characteristics at baseline by intervention condition (N=1369).^a^

Characteristics			Total sample (N=1369)		Basic (n=560)		Enhanced (n=574)		E-pamphlet (n=235)
Age (years), mean (SD)			22.30 (2.32)		22.24 (2.32)		22.37 (2.34)		22.26 (2.29)
**Gender identity, n (%)**									
	Male	382 (27.9)		74 (31.5)		155 (27.7)		153 (26.7)	
	Female	949 (69.3)		156 (66.4)		390 (69.6)		403 (70.2)	
	Transgender male	35 (2.6)		4 (1.7)		15 (2.7)		16 (2.8)	
	Transgender female	3 (0.2)		1 (0.4)		0 (0.0)		2 (0.3)	
**Race and ethnicity, n (%)**									
	White non-Hispanic	1141 (83.3)		471 (84.7)		476 (83.5)		194 (83.3)	
	Other	218 (15.9)		85 (15.3)		94 (16.5)		39 (16.7)	
**Employment status, n (%)**									
	0 hours (does not work)	123 (9.0)		45 (8.0)		58 (10.1)		20 (8.5)	
	1-29 hours (part time)	631 (46.1)		254 (45.4)		265 (46.2)		112 (47.7)	
	>30-39 hours (approximately full time or more)	615 (44.9)		261 (46.6)		251 (43.7)		103 (43.8)	
**Sexual orientation, n (%)**									
	Heterosexual	999 (73.0)		404 (72.1)		426 (74.2)		169 (71.9)	
	Gay, lesbian, bisexual, other	343 (25.1)		144 (25.7)		138 (24.0)		61 (26.0)	
**Education level, n (%)**									
	Less than high school or not yet graduated	35 (2.6)		15 (2.7)		12 (2.1)		8 (3.4)	
	High school graduate or general educational diploma	107 (7.8)		43 (7.7)		42 (7.3)		22 (9.4)	
	Some college	757 (55.3)		328 (58.6)		311 (54.2)		118 (50.2)	
	Graduated college	397 (29.0)		146 (26.1)		171 (29.8)		80 (34.0)	
	Graduate degree or higher	73 (5.3)		28 (5.0)		38 (6.6)		7 (3.0)	
**Annual income (US $), n (%)**									
	0-15,000	649 (47.4)		254 (47.2)		288 (51.0)		107 (46.3)	
	>15,000	685 (51.3)		284 (52.8)		277 (49.0)		124 (53.7)	
**Current climate, n (%)**									
	Northern	1021 (74.6)		422 (75.4)		425 (74.3)		174 (74.4)	
	Southern or Hawaii	345 (25.2)		138 (24.6)		147 (25.7)		60 (25.6)	
**Cohabitation status, n (%)**									
	Lived alone	123 (9.0)		47 (8.4)		54 (9.4)		22 (9.4)	
	Lived with someone else	1185 (86.6)		485 (86.8)		493 (86.0)		207 (88.1)	
	Lived alone and with someone else (lived with most)	59 (4.3)		27 (4.8)		26 (4.5)		6 (2.6)	

^a^Missing data were excluded from Table 2. The greatest percentage of missing data for any variable was 2.6% (income).

### Primary and Secondary Outcomes

Mean scores at baseline on primary outcomes were low, with an average score of 1.72 (SD 0.49) for overall sun protection, an average score of 1.48 (SD 0.84) for habitual sun protection on scales from 0 to 4, and an average of 0.72 (SD 1.35) for overall UVR exposure on a scale from 0 to 30 in the last month (Table 3). Baseline values on secondary outcomes were also low, with 11.2% (n=154) reporting at least 1 skin self-exam in the last 3 months, 10.4% (n=143) reporting a physician skin exam in the last 12 months, 31.6% (n=416) reporting wearing sunscreen on their body at least once in the last month, 6.9% (n=94) reporting indoor tanning at least once in the last month, yet 23.4% (n=321) reporting at least 1 sunburn in the last month (Table 4).

**Table 3:** Summary descriptive statistics for primary outcomes by intervention and time point (unadjusted).

Primary outcomes (for the last month) and time point (weeks)		Total, mean (SD)	Basic (n=560), mean (SD)	Enhanced (n=574), mean (SD)	E-pamphlet (n=235), mean (SD)	*P* value
**Sun protection (0=never to 4=almost always)**						
	Baseline	1.72 (0.49)	1.73 (0.46)	1.70 (0.51)	1.74 (0.50)	.51
	Week 04	1.82 (0.55)	1.80 (0.54)	1.86 (0.58)	1.77 (0.49)	.09
	Week 12	1.96 (0.57)	1.96 (0.58)	2.01 (0.58)	1.85 (0.53)	.004
	Week 24	2.09 (0.58)	2.10 (0.59)	2.12 (0.59)	2.00 (0.54)	.04
	Week 52	1.98 (0.59)	1.98 (0.62)	2.02 (0.58)	1.89 (0.54)	.02
**Habitual protection (0=never to 4=almost always)**						
	Baseline	1.48 (0.84)	1.47 (0.86)	1.49 (0.83)	1.46 (0.84)	.90
	Week 04	1.70 (0.90)	1.69 (0.91)	1.75 (0.91)	1.60 (0.84)	.10
	Week 12	1.86 (0.95)	1.88 (0.92)	1.90 (0.98)	1.73 (0.92)	.08
	Week 24	1.95 (0.94)	1.99 (0.91)	2.00 (0.94)	1.78 (0.99)	.02
	Week 52	2.13 (0.97)	2.16 (0.98)	2.14 (0.97)	2.04 (0.97)	.38
**UVR exposure (number of days)**						
	Baseline	0.72 (1.35)	0.74 (1.34)	0.75 (1.45)	0.60 (1.09)	.20
	Week 04	0.44 (1.00)	0.43 (0.99)	0.43 (1.01)	0.46 (1.00)	.92
	Week 12	0.70 (1.20)	0.66 (1.12)	0.71 (1.20)	0.79 (1.36)	.43
	Week 24	1.26 (1.57)	1.31 (1.67)	1.19 (1.47)	1.33 (1.56)	.36
	Week 52	0.38 (0.86)	0.39 (0.95)	0.40 (0.80)	0.34 (0.77)	.68

**Table 4:** Summary descriptive statistics for secondary outcomes by intervention and time point (unadjusted).

Secondary outcomes and visit (weeks)		Total, n (%)	Basic (n=560), n (%)	Enhanced (n=574), n (%)	E-pamphlet (n=235), n (%)	*P* value
**Skin self-exam (≥1 time in last 3 months)**						
	Baseline	154 (11.2)	65 (11.6)	70 (12.2)	19 (8.1)	.23
	Week 12	206 (17.4)	91 (19.1)	91 (18.2)	24 (11.7)	.052
	Week 24	254 (21.6)	111 (22.9)	107 (22.0)	36 (17.4)	.26
	Week 52	272 (23.8)	109 (23.8)	130 (26.8)	33 (16.4)	.01
**Physician skin exam (≥1 time in last 12 months)**						
	Baseline	143 (10.4)	61 (10.9)	64 (11.1)	18 (7.7)	.31
	Week 04	137 (11.6)	60 (12.6)	56 (11.1)	21 (10.1)	.61
	Week 12	171 (15.5)	79 (16.6)	71 (14.2)	21 (10.2)	.09
	Week 24	185 (15.7)	75 (15.5)	80 (16.5)	30 (14.5)	.80
	Week 52	229 (20.0)	101 (22.1)	92 (19.0)	36 (17.9)	.35
**Sunscreen on body (≥1 time in last month)**						
	Baseline	416 (31.6)	165 (30.4)	181 (33.3)	70 (30.7)	.56
	Week 04	375 (33.1)	143 (31.3)	168 (35.3)	64 (32.3)	.41
	Week 12	517 (45.6)	211 (45.6)	233 (49.2)	73 (36.9)	.01
	Week 24	857 (73.6)	356 (74.6)	355 (74.0)	146 (70.5)	.52
	Week 52	456 (41.6)	179 (40.8)	199 (43.1)	78 (40.0)	.69
**Indoor tanning (≥1 time in last month)**						
	Baseline	94 (6.9)	39 (7.0)	45 (7.8)	10 (4.3)	.19
	Week 04	55 (4.6)	23 (4.8)	25 (5.0)	7 (3.4)	.64
	Week 12	54 (4.6)	21 (4.4)	23 (4.6)	10 (4.9)	.97
	Week 24	39 (3.3)	17 (3.5)	17 (3.5)	5 (2.4)	.73
	Week 52	34 (3.0)	17 (3.7)	15 (3.1)	2 (1.0)	.16
**Sunburn (≥1 time in last month)**						
	Baseline	321 (23.4)	145 (25.9)	124 (21.6)	52 (22.1)	.20
	Week 04	202 (17.0)	81 (17.0)	79 (15.7)	42 (20.3)	.34
	Week 12	357 (30.2)	141 (29.6)	157 (31.4)	59 (28.6)	.72
	Week 24	567 (48.2)	238 (49.2)	224 (46.1)	105 (50.7)	.45
	Week 52	167 (14.6)	68 (14.8)	74 (15.3)	25 (12.4)	.62

### Intervention Effects on Primary Outcomes

**Figure 2:**
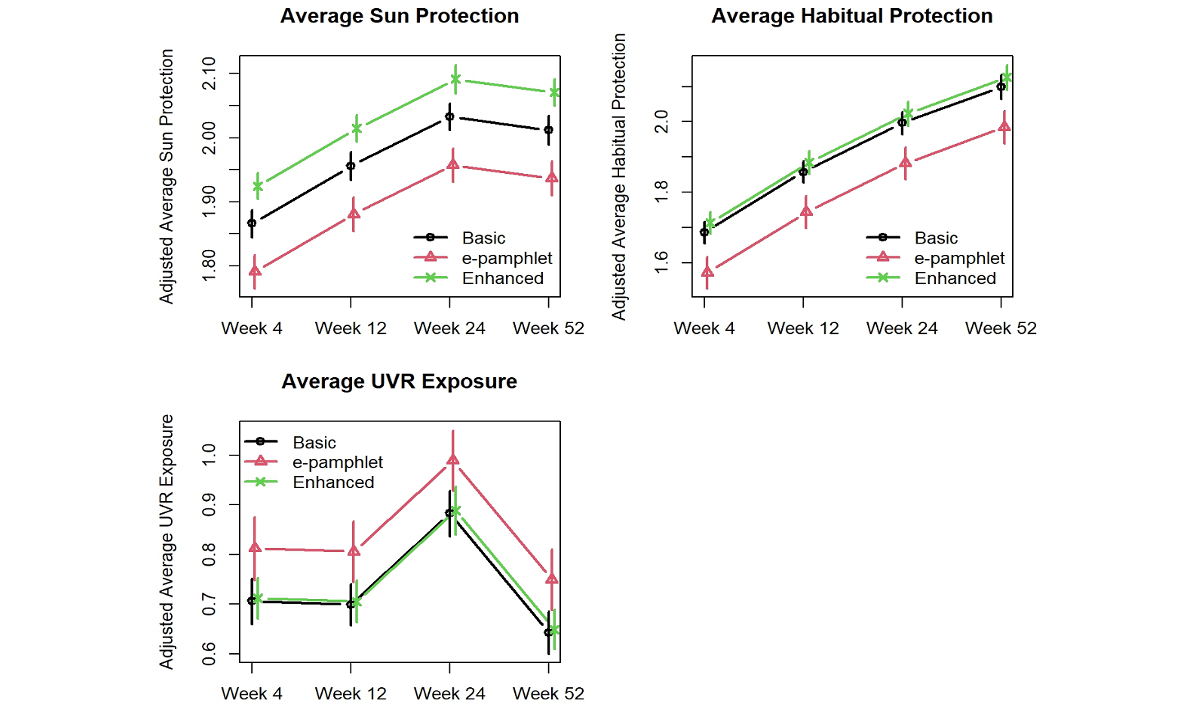
Intervention effects on primary outcomes. UVR: Ultraviolet radiation.

Figure 2 shows the error bar plots of the changes from Baseline of the primary outcomes. As the interactions of intervention and time were not significant for any primary outcome (*P*≥.23), they were not included in the final models. Overall sun protection in the e-pamphlet group was statistically significantly lower than that in the basic or the enhanced group (*P*=.007 and <.001 with Cohen *d* of 0.209 and 0.376, respectively). In addition, sun protection in the enhanced group was statistically significantly higher than that in the basic group (*P*=.01, *d*=0.149). Habitual sun protection in the e-pamphlet group was statistically significantly lower than that in the basic or the enhanced group (*P*=.02 and .004 with *d* of 0.186 and 0.224, respectively); there was no difference between the basic and the enhanced groups (*P*=.46). All groups reported increased UVR exposure during the summer, and UVR exposure in the e-pamphlet group was greater than that in the basic or the enhanced group, but these differences did not reach statistical significance (*P*=.07 and .09, respectively) In addition, there was no significant difference in UVR between the basic and enhanced groups (*P*=.89).

### Intervention Effects on Secondary Outcomes

**Figure 3:**
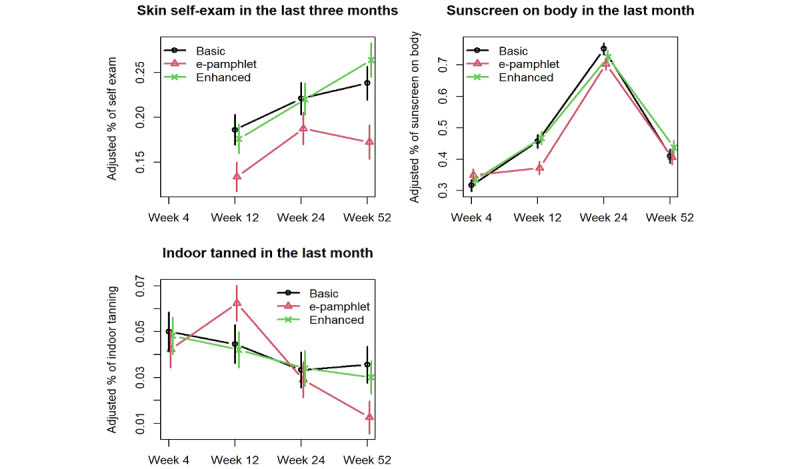
Intervention effects on secondary outcomes.

Figure 3 shows the error bar plots of the adjusted proportions for secondary outcomes. For the adjusted proportions of skin self-exam in the past 3 months, the basic group had a significantly higher proportion of individuals who reported doing at least 1 skin self-exam than those in the e-pamphlet group at week 52 (*P*=.04). The proportion for the enhanced group was also significantly higher than that for the e-pamphlet group at week 52 (*P*=.004). For the adjusted proportion of individuals who use sunscreen on their body, which increased for all groups during the summer, the proportions for both the basic and enhanced groups were significantly higher than that for the e-pamphlet group at week 12 (*P*=.03 and .01, respectively). For the adjusted percent of indoor tanning in the past month, the basic group had a significantly higher proportion of individuals who reported indoor tanning than the e-pamphlet group at week 52 (*P*=.048). All other group comparisons from week 4 to week 52 were not statistically significant.

## Discussion

This study was a relatively large national hybrid type II effectiveness-implementation RCT of the first, if not only, existing fully digital behavioral intervention that has been demonstrated to modify skin cancer risk behaviors among healthy young adults in the United States at risk of skin cancer recruited from the community with longitudinal follow-ups over the course of a year. Compared with the e-pamphlet, the active interventions increased overall sun protection and habitual sun protection and decreased overall UVR exposure as expected. Similarly, the active interventions also increased sunscreen use worn on the body at the 3-month follow-up (end of summer or early fall) and skin self-examination at 1 year compared with the e-pamphlet. However, the enhanced intervention only differentially improved overall sun protection but did not improve other behaviors more than the basic intervention did. Other outcome effects (eg, for sunburn) were nonsignificant.

Our results suggest that all the automated digital interventions that can be delivered via a smartphone had a significant impact on UVR exposure and protection behaviors as well as skin self-examination. Overall sun protection increased over time for all groups, suggesting that a simple digital pamphlet can produce some behavioral change. However, participants in the e-pamphlet group did engage in (nonstatistically significantly) more UVR exposure at all follow-ups compared with the other groups, so content on UVR exposure may need to be intensified in future e-pamphlets. Additionally, the content on skin self-exam in the active interventions was relatively minimal, which is promising for future interventions addressing this particular behavior. However, the basic intervention increased the physician skin exam significantly in the prior study [58] but not this one.

UVR exposure and sun protection patterns and intervention effects differed by outcome and across time as well as compared with our prior study of the original basic intervention, which demonstrated stronger early effects [58]. Overall sun protection and skin self-exam behavior showed more linear trends over time. UVR exposure, sunscreen use, and sunburns appeared to be more closely associated with changes in seasons. Sunburn patterns were most similar to sunscreen use on the body as opposed to UVR exposure, suggesting that interventions addressing long-term sunscreen use (vs UVR exposure or tanning) may be beneficial to reduce sunburns. Although intervention effects on overall sun protection were fairly stable through 1 year, the differences in the impact of the active interventions on wearing sunscreen compared with e-pamphlets were only significant at 12 weeks and not beyond. Current controversies about potential advantages and disadvantages of mineral, chemical, and hybrid sunscreens for humans and the environment add to the challenges of refining content for such behavioral interventions [99]. The overall proportion of the sample experiencing sunburn decreased from week 4 to 24, but the effects of the interventions did not differ significantly despite the fact that the basic intervention had been differentially efficacious at 12 weeks in the prior study. Indoor tanning decreased overall from week 4 to week 52. However, a higher proportion of the basic group reported indoor tanning at 52 weeks compared with the other intervention conditions. This is a surprising finding and difficult to interpret especially because the material on indoor tanning was similar for both active interventions and was addressed briefly in the e-pamphlet intervention. In the prior study, indoor tanning did not differ by intervention group.

Goal setting and changes in attitudes addressed in the IM mediator messages were associated with improved outcomes in the prior study. However, compared with the other intervention conditions, the enhancements that were made to the enhanced intervention including enhanced goal setting, embedded incentives, and ongoing mediator messages and video or news updates had a significantly greater impact only on overall sun protection behaviors. There were no other significant differences in effects between the basic and enhanced interventions. This may be due in part to the content of these enhancements. For example, the incentives were mostly related to sun protection (eg, discounts on sunscreen, protective clothing, hats, and sunglasses). Additionally, the incentives were discounts rather than actual money or protective items. Although participants were paid for completing surveys, we do not know whether they used the discounts to purchase sun safety items, which were relatively expensive even with the discounts. All the enhancements were intended to increase intervention engagement over time and thus intervention effects. However, maintaining engagement with digital interventions past an initial exposure is an ongoing challenge for the field. A subsequent paper from this study will explore attitudes and intervention engagement to determine their associations with outcomes. A future noninferiority trial could also assess whether the basic and enhanced interventions produce similar engagement and effects on sun protection if the updated news and video features are removed from the enhanced intervention, thus making it fully automated. Future studies may benefit from more explicit consideration and evaluation of constructs and models related to (digital) intervention engagement behaviors versus health behavior change interventions, either separately or integrated together. Another important difference between the current and prior trial is that the current trial compared 3 interventions, one of which had already been found to be efficacious, which may have resulted in a ceiling effect. The prior study included an assessment-only condition, so it may have been able to demonstrate greater effects.

The limitations of the study include the convenience sample, largely from northern US climates, and that not all participants were recruited during the spring and summer months when risk behaviors are highest. The proportion of recent indoor tanners was relatively low and may limit conclusions related to this outcome. In addition to addressing these limitations, future work should focus on interventions that are less costly to build initially, for example, by using existing social media platforms for the actual interventions. The use of pragmatic trials or more complex, yet potentially efficient, study designs such as factorials or adaptive designs might help determine which aspects of interventions are most cost-effective (eg, goal setting, mediator messages, incentives, or ongoing content updates) for which specific behaviors (eg, sunburn or indoor tanning) and populations. Although some intervention effects seemed to last the full year, more engaging and intense “boosters” may enhance the longitudinal impact. Initial and ongoing engagement and response quality remain challenges for the field.

In conclusion, the strengths of the study include the relatively large national RCT of one of the few digital sun safety interventions for the at-risk young adult population, featuring social media recruitment and longitudinal follow-ups over the course of a year. The automated interventions modified UVR exposure, sunscreen use, overall sun protection, and skin self-examination. However, the effects of the enhanced intervention differed from those of the basic intervention only on sun protection, not other outcomes.

## Appendix

Multimedia Appendix 1 CONSORT-eHEALTH checklist (V 1.6.2).

## Abbreviations

BRAT: Brief Risk Assessment Tool
CONSORT: Consolidated Standards of Reporting Trials
DQS: data quality score
IM: Integrated Model of Behavioral Prediction
MI: multiple imputation
RCT: randomized controlled trial
UVR: UV radiation

## Data Availability

The data set generated during this study is available from the corresponding author upon reasonable request.
