# Supplementary material for: Digital Interventions to Modify Skin Cancer Risk Behaviors in a National Sample of Young Adults: Randomized Controlled Trial
Source: J Med Internet Res. 2024 Jul 2;26:e55831. doi: 10.2196/55831 (PMC11252624; doi:10.2196/55831)
Supplement: Multimedia Appendix 1 [file jmir_v26i1e55831_app1.pdf]

CONSORT-EHEALTH Checklist V1.6.2 Report	Manuscript Number	55831
**(based on CONSORT-EHEALTH V1.6), available at [http://tinyurl.com/consort-ehealth-v1-6].**		
**Date completed**		
1/26/2024 8:33:18		
**by**		
Carolyn Heckman		
TITLE		
**1a-i) Identify the mode of delivery in the title**		
**1a-ii) Non-web-based components or important co-interventions in title**		
Yes, "Digital Interventions"...		
**1a-iii) Primary condition or target group in the title**		
ABSTRACT		
**1b-i) Key features/functionalities/components of the intervention and comparator in the METHODS section of the ABSTRACT**		
**1b-ii) Level of human involvement in the METHODS section of the ABSTRACT**		
Yes: "This study was a Hybrid Type II Effectiveness-Implementation randomized controlled trial of two active interventions, a digital skin cancer risk reduction intervention (UV4.Me [Basic]) compared to an enhanced version (UV4.Me2 [Enhanced]), and an e-pamphlet… Enhancements to encourage intervention engagement and behavior change included more comprehensive goal-setting activities, ongoing proactive messaging related to previously established mediators (e.g., self-efficacy) of UVR exposure and protection, embedded incentives for module completion, and ongoing news and video updates."		
**1b-iii) Open vs. closed, web-based (self-assessment) vs. face-to-face assessments in the METHODS section of the ABSTRACT**		
**1b-iv) RESULTS section in abstract must contain use data**		
**1b-v) CONCLUSIONS/DISCUSSION in abstract for negative trials**		
INTRODUCTION		
**2a-i) Problem and the type of system/solution**		
**2a-ii) Scientific background, rationale: What is known about the (type of) system**		
Yes: "Our goal was to improve the impact of the Basic Intervention on sun protection, UVR exposure, and engaging in skin cancer examination. To improve effects of the Basic Intervention, we added several key interactive features/strategies suggested by prior participants, our data, and supported by the literature (i.e., by creating a mobile version, adding incentives embedded in the intervention, an enhanced goal-oriented feature, ongoing email and text messages related to previously identified mediators of behavior change, and ongoing news and video updates)."		
METHODS		
**3a) CONSORT: Description of trial design (such as parallel, factorial) including allocation ratio**		
**3b) CONSORT: Important changes to methods after trial commencement (such as eligibility criteria), with reasons**		
Yes: "We hypothesized that effectiveness and maintenance outcomes would be best for Enhanced, next best for the original Basic condition, and worst for a... control group..."		
**3b-i) Bug fixes, Downtimes, Content Changes**		
**4a) CONSORT: Eligibility criteria for participants**		
It is stated in the abstract and introduction section that the study conducted was a "Hybrid Type II Dissemination-Effectiveness Trial." The allocation ratio is stated in the methods, "A computerized program was created to randomize participants on a 2:2:1 basis in blocks of 10 to either Basic, Enhanced (described below), or [control intervention]..."		
**4a-i) Computer / Internet literacy**		
**4a-ii) Open vs. closed, web-based vs. face-to-face assessments:**		
**4a-iii) Information giving during recruitment**		
The study was web-based and closed, where participants had to screen into participation online, as described in the Methods, "recruitment of study participants was conducted through paid advertising on Facebook, Instagram, and Twitter and unpaid social media posts between September, 2018 and April, 2019... Individuals were then instructed to create an account on the sign-up webpage with a phone number, email, and password [if they clicked the ad]. Once an individual indicated their interest and created a study account, they were automatically directed to complete a brief eligibility screener. If eligible, individuals were invited to complete the online informed consent form." Because we were recruiting a large sample through social media advertising, data quality was important to assessed, as described in the methods...		
**4b) CONSORT: Settings and locations where the data were collected**		
Changes were not made to the trial or eligibility criteria after study commencement.		
**4b-i) Report if outcomes were (self-)assessed through online questionnaires**		
**4b-ii) Report how institutional affiliations are displayed**		
Participants completed online self-assessment surveys, as described in the methods section. It states, "Upon consent, participants were directed to the 10-minute online baseline survey. If enrollees completed the baseline survey… Participants were invited by email, text message, and/or telephone if necessary to complete online 10-15 minute follow-up surveys at 4,12, 24, and 52 weeks."		
**5) CONSORT: Describe the interventions for each group with sufficient details to allow replication, including how and when they were actually administered**		
**5-i) Mention names, credential, affiliations of the developers, sponsors, and owners**		
**5-ii) Describe the history/development process**		
**5-iii) Revisions and updating**		
**5-iv) Quality assurance methods**		
**5-v) Ensure replicability by publishing the source code, and/or providing screenshots/screen-capture video, and/or providing flowcharts of the algorithms used**		
**5-vi) Digital preservation**		
**5-vii) Access**		

**5-viii) Mode of delivery, features/functionalities/components of the intervention and comparator, and the theoretical framework**		
Yes: “Upon consent, participants were directed to the 10-minute online baseline survey. If enrollees completed the baseline survey, they were automatically directed to their intervention condition and received a $5 electronic gift card… Participants were informed that the total study incentives would be up to $120 for completing all five online surveys over the course of twelve months, plus periodic gift card raffles.”<br>“Individuals were then instructed to create an account on the sign-up webpage with a phone number, email, and password. Once an individual indicated their interest and created a study account, they were automatically directed to complete a brief eligibility screener.”		
**5-ix) Describe use parameters**		
“UV4.me (Basic): The IM informed the development of the Basic Intervention, for example by focusing on modification of attitudes, beliefs, perceived norms and self-efficacy, as well as intentions and behaviors. The Basic Intervention, described elsewhere in more detail [55], is targeted to YAs, individually tailored, and includes interactive, multimedia, and goal-setting components. It includes 12 modules with content related to a specific topic important in terms of risk or protective behaviors...<br>UV4.me2 (Enhanced): New features/strategies were chosen based on participant feedback from the original UV4.me (e.g., strategies suggested to make Basic more interactive), our data (e.g., number of mobile users who tried to access Basic), and evidence-based literature and reviews/models of effective e-Health interventions [61, 62] and implementation strategies [63-65]. Several enhancements were expected to improve recruitment and representativeness, engagement, and behavioral outcomes: 1. Mobile site [compatible], 2. [Personal] goal setting [feature], 3. Mediator messages [sent via email and/or text message], 4. Incentives, [like coupons for engagement], 5. Ongoing news updates and video library.<br>Control Condition: E-Pamphlet: A free online American Cancer Society pamphlet on skin cancer prevention and early detection [60] was used as the control condition and was distributed on a non-interactive webpage.”		
**5-x) Clarify the level of human involvement**		
**5-xi) Report any prompts/reminders used**		
**5-xii) Describe any co-interventions (incl. training/support)**		
Participants in the Enhanced intervention condition were sent mediator messages to prompt motivation to engage with certain features of the intervention or to engage in health promoting behaviors. “Goal setting. Goal setting addresses several aspects of the IM, e.g., intentions, environmental constraints, self-efficacy, and behavior. Users were given the option to commit to a behavioral goal relevant to each module... They were given tips on goal setting..., asked to select from a list of potential reasons the goal might be important to them,... strategies to assist them,... note their progress,... and challenges,... over time, see their goals summarized on a page called MyGoals, and receive motivating email/text messages,... and online feedback... Email and/or text messages were sent to participants based on IM constructs that were found to mediate UVR exposure and sun protection outcomes in the prior study, e.g., knowledge and self-efficacy. Enhanced participants were sent six mediator messages with decreasing frequency over the course of the year"<br>Participants were periodically sent reminder messages via email and text message to login to their assigned interventions and complete surveys.		
**6a) CONSORT: Completely defined pre-specified primary and secondary outcome measures, including how and when they were assessed**		
“Participants eligible for the study were 18-25 years old, or 19-25 if in Alabama or Nebraska,.. English speakers, living in a US state or D.C., had at least weekly internet access, did not have a personal history of skin cancer, and did not report “always” protecting their skin from the sun when outdoors during warm weather. Participants also responded to... items related to phenotypic and/or behavioral risk factors (e.g., fair skin, history of sunburns) that indicated moderate to high risk of developing skin cancer... [78].”		
**6a-i) Online questionnaires: describe if they were validated for online use and apply CHERRIES items to describe how the questionnaires were designed/deployed**		
**6a-ii) Describe whether and how “use” (including intensity of use/dosage) was defined/measured/monitored**		
**6a-iii) Describe whether, how, and when qualitative feedback from participants was obtained**		
**6b) CONSORT: Any changes to trial outcomes after the trial commenced, with reasons**		
Yes: “… a study-specific landing webpage or sign-up webpage… Once an individual indicated their interest and created a study account, they were... directed to complete a brief eligibility screener. If eligible, individuals were invited to complete the online informed consent form. Upon consent, participants were directed to the 10-minute online baseline survey.”		
**7a) CONSORT: How sample size was determined**		
**7a-i) Describe whether and how expected attrition was taken into account when calculating the sample size**		
**7b) CONSORT: When applicable, explanation of any interim analyses and stopping guidelines**		
Yes: “Primary Outcomes: Skin cancer-related behavioral outcomes were assessed at baseline and four follow-up time-points: 4,12, 24, and 52 weeks. We assessed sun protection frequency in the last month... how many days in the past month they experienced various types of UVR exposure... [22, 82, 83]. We adapted the 4-items from the Habit Automaticity subscale of the Self-Report Habit Index to measure behavior patterns associated with the formation of a habit... [84] Secondary Outcomes: Specific items including sunburns and indoor tanning were considered secondary outcomes since sunburns and indoor tanning are unique risk factors for skin cancers, and these occur less frequently than sun exposure and protection in general. Participants were also asked about whether they had been screened for skin cancer by a healthcare provider or by themselves or a partner.”		
**8a) CONSORT: Method used to generate the random allocation sequence**		
There have not been any changes to the trial outcomes after the trial commenced.		
**8b) CONSORT: Type of randomisation; details of any restriction (such as blocking and block size)**		
This was a low-risk behavioral science trial, so there were no stopping guidelines outside of standard IRB requirements. There were no interim analyses of the outcomes.		
**9) CONSORT: Mechanism used to implement the random allocation sequence (such as sequentially numbered containers), describing any steps taken to conceal the sequence until interventions were assigned**		
The random allocation sequence was created using “a computerized program.”		
**10) CONSORT: Who generated the random allocation sequence, who enrolled participants, and who assigned participants to interventions**		
"A computerized program was created to randomize participants on a 2:2:1 basis in blocks of 10 to either Basic, Enhanced… [control]…”		
**11a) CONSORT: Blinding - If done, who was blinded after assignment to interventions (for example, participants, care providers, those assessing outcomes) and how**		
**11a-i) Specify who was blinded, and who wasn’t**		
**11a-ii) Discuss e.g., whether participants knew which intervention was the “intervention of interest” and which one was the “comparator”**		
Participants were not blind to their conditions, as the conditions were the web interventions that they interacted with. The study team and outcome assessors were not blind to the conditions either. It is mentioned in the methods that participants were considered having “suspicious” data if they were “reporting that they knew someone else in the study [and/or they were] reporting on features only included in another condition…” etc.		
**11b) CONSORT: If relevant, description of the similarity of interventions**		
"A computerized program was created to randomize participants on a 2:2:1 basis in blocks of 10 to either Basic, Enhanced (described below), or a non-interactive skin cancer prevention educational webpage...”		
**12a) CONSORT: Statistical methods used to compare groups for primary and secondary outcomes**		
We do not include access to the full study protocol. However, the study is registered with clinicaltrials.gov and additional information can be found there.		
**12a-i) Imputation techniques to deal with attrition / missing values**		
**12b) CONSORT: Methods for additional analyses, such as subgroup analyses and adjusted analyses**		
All interventions are described in detail in text of the paper. Description of the similarities of the interventions is not relevant.		
RESULTS		
**13a) CONSORT: For each group, the numbers of participants who were randomly assigned, received intended treatment, and were analysed for the primary outcome**		
**13b) CONSORT: For each group, losses and exclusions after randomisation, together with reasons**		

"We calculated total costs of each intervention arm from the program perspective by summing costs reported in monthly submissions of cost data collection instruments… Intervention delivery costs included in the analysis were fixed as they did not vary with intervention enrollment... Specifically, we estimated the cost required to achieve one additional skin self-exam in each intervention group relative to e-pamphlet. We calculated this cost-effectiveness ratio as the difference in intervention delivery costs (UV4me vs. e-pamphlet and UV4me2 vs. e-pamphlet) divided by the difference in changes from baseline to follow-up on the probability of conducting a skin self-exam in the past three months."		
**13b-i) Attrition diagram**		
**14a) CONSORT: Dates defining the periods of recruitment and follow-up**		
The number of participants randomly assigned to each intervention group are displayed in Table 2 in the results section.		
**14a-i) Indicate if critical "secular events" fell into the study period**		
**14b) CONSORT: Why the trial ended or was stopped (early)**		
The number of participants lost or excluded from follow up are included in the CONSORT flow diagram. Details of why participants were excluded are detailed in the results.		
**15) CONSORT: A table showing baseline demographic and clinical characteristics for each group**		
"Briefly, recruitment of study participants was conducted through paid advertising on Facebook, Instagram, and Twitter and unpaid social media posts between September, 2018 and April, 2019." The dates of the participant follow up surveys are written in the results section of the paper: "Of the 1369 who were randomized to one of the three intervention conditions, the proportion of completed surveys at follow up were 1158 (84.6%) at 4 weeks, 1182 (86.3%) at 12 weeks, 1177 (86.0%) at 26 weeks, and 1144 (83.6%) at 52 weeks. These completion rates were similar across the three intervention conditions."		
**15-i) Report demographics associated with digital divide issues**		
**16a) CONSORT: For each group, number of participants (denominator) included in each analysis and whether the analysis was by original assigned groups**		
**16-i) Report multiple "denominators" and provide definitions**		
**16-ii) Primary analysis should be intent-to-treat**		
Yes, the Ns for the study are stated in the results and in the CONSORT flow diagram.		
**17a) CONSORT: For each primary and secondary outcome, results for each group, and the estimated effect size and its precision (such as 95% confidence interval)**		
The trial was not ended or stopped early so this was not mentioned in the manuscript.		
**17a-i) Presentation of process outcomes such as metrics of use and intensity of use**		
**17b) CONSORT: For binary outcomes, presentation of both absolute and relative effect sizes is recommended**		
See table 1 for details.		
**18) CONSORT: Results of any other analyses performed, including subgroup analyses and adjusted analyses, distinguishing pre-specified from exploratory**		
See Figures 2 and 3 for details.		
**18-i) Subgroup analysis of comparing only users**		
**19) CONSORT: All important harms or unintended effects in each group**		
No, our paper does not address this.		
**19-i) Include privacy breaches, technical problems**		
**19-ii) Include qualitative feedback from participants or observations from staff/researchers**		
DISCUSSION		
**20) CONSORT: Trial limitations, addressing sources of potential bias, imprecision, multiplicity of analyses**		
**20-i) Typical limitations in ehealth trials**		
**21) CONSORT: Generalisability (external validity, applicability) of the trial findings**		
**21-i) Generalizability to other populations**		
**21-ii) Discuss if there were elements in the RCT that would be different in a routine application setting**		
**22) CONSORT: Interpretation consistent with results, balancing benefits and harms, and considering other relevant evidence**		
**22-i) Restate study questions and summarize the answers suggested by the data, starting with primary outcomes and process outcomes (use)**		
**22-ii) Highlight unanswered new questions, suggest future research**		
Yes: "Compared to the e-pamphlet, the active interventions increased overall sun protection and habitual sun protection and decreased overall UVR exposure. The active interventions also increased sunscreen use worn on the body at three-month follow-up (end of summer/early fall) and skin self-examination at one year compared to the e-pamphlet. The Enhanced Intervention differentially improved overall sun protection but not other behaviors compared to the Basic Intervention. Other outcome effects (e.g., for sunburn) were non-significant. After initial development, the active interventions were similar in cost per person with increasing numbers of participants and incurred reasonable costs to achieve an additional SSE compared with e-pamphlet."		
Other information		
**23) CONSORT: Registration number and name of trial registry**		
Subgroup intervention analyses are included in the results section.		
**24) CONSORT: Where the full trial protocol can be accessed, if available**		
This is a low-risk intervention study, so there were no harms or unintended effects to report for any of the groups.		
**25) CONSORT: Sources of funding and other support (such as supply of drugs), role of funders**		
The registration numbers are stated in the Abstract section of the manuscript:<br>"Trial Registration: ClinicalTrials.gov ID NCT03313492"		
**X26-i) Comment on ethics committee approval**		
**x26-ii) Outline informed consent procedures**		
**X26-iii) Safety and security procedures**		
**X27-i) State the relation of the study team towards the system being evaluated**		
